# Modified Local Linear Estimators in Partially Linear Additive Models with Right-Censored Data Based on Different Censorship Solution Techniques

**DOI:** 10.3390/e25091307

**Published:** 2023-09-07

**Authors:** Ersin Yılmaz, Dursun Aydın, S. Ejaz Ahmed

**Affiliations:** 1 Department of Statistics, Mugla Sıtkı Kocman University, Mugla 48000, Turkey; duaydin@mu.edu.tr; 2 Department of Mathematics and Statistics, Brock University, St. Catharines, ON L2S 3A1, Canada; sahmed5@brocku.ca

**Keywords:** partially linear additive models, local linear regression, right-censored data, synthetic data, kNN imputation

## Abstract

This paper introduces a modified local linear estimator (LLR) for partially linear additive models (PLAM) when the response variable is subject to random right-censoring. In the case of modeling right-censored data, PLAM offers a more flexible and realistic approach to the estimation procedure by involving multiple parametric and nonparametric components. This differs from the widely used partially linear models that feature a univariate nonparametric function. The LLR method is employed to estimate unknown smooth functions using a modified backfitting algorithm, delivering a non-iterative solution for the right-censored PLAM. To address the censorship issue, three approaches are employed: synthetic data transformation (ST), Kaplan–Meier weights (KMW), and the kNN imputation technique (kNNI). Asymptotic properties of the modified backfitting estimators are detailed for both ST and KMW solutions. The advantages and disadvantages of these methods are discussed both theoretically and practically. Comprehensive simulation studies and real-world data examples are conducted to assess the performance of the introduced estimators. The results indicate that LLR performs well with both KMW and kNNI in the majority of scenarios, along with a real data example.

## 1. Introduction

Partially linear models (PLMs) have gained considerable attention in the field of survival analysis, especially for modeling right-censored data. The flexibility and capability of PLMs to capture both parametric and nonparametric components make them a favored choice for analyzing survival data with complex relationships. The classical PLM is expressed as follows for completely observed data with a sample size n:

(1) $$y_{i}=x_{i}^{T}\beta +f\left(t_{i}\right)+\varepsilon_{i},\;1\leq i\leq n\;$$

where $y_{i}$’s are the completely observed response values (or lifetimes in survival analysis), $x_{i}\in R^{n\times p}$ are the parametric covariates, $\beta ={\left(\beta_{1},\ldots ,\beta_{p}\right)}^{T}$ denotes the $\left(p\times 1\right)$ dimensional vector of regression coefficients, and $f\left(.\right)$ is the univariate unknown smooth function to be estimated based on the values of the nonparametric covariate $t_{i}$’s. Finally, $\varepsilon_{i}$’s are the random error terms with $\left(i\right)\;\varepsilon_{i}\sim \;N\left(0,\sigma_{\varepsilon }^{2}\right)\;\mathrm{and}\;\left(ii\right)\;Cov\left(\varepsilon_{i},x_{i}\right)=0,\;\left(iii\right)\;E [\varepsilon_{i}|x_{i},t_{i}]=0$. Without censored data, model (1) has been studied by many researchers, and some of the notable studies include [1,2], among others. Additionally, ref. [3] proposed the local linear regression (LLR) estimation for model (1). In the right-censored case, the response variable, $y_{i}$, is incompletely observed and censored from the right by random censoring variable ${\left\{c_{i}\right\}}_{i=1}^{n}$ under the assumption that $x_{i}$ and $t_{i}$ are completely observed. Accordingly, the censoring mechanism and some new variables can be obtained as follows:

(2) $$z_{i}=\min\left(y_{i},c_{i}\right)\;\mathrm{with}\;\delta_{i}=\left\{\begin{array}{c} 0,\;if\;y_{i}\;is\;censored\;\left(y_{i}>c_{i}\right)\\ 1,\;if\;y_{i}\;is\;uncensored\;\left(y_{i}\leq c_{i}\right) \end{array}\right.\;$$

where $z_{i}$ denotes the incompletely observed response variable with the censoring indicator $\delta_{i}$. Thus, instead of $y_{i}$, data pairs $\left\{z_{i},\delta_{i}\right\}$ are used in the modeling procedure. There are several important studies on the estimation of model (1) under right-censored data, as given in (2), such as refs. [4,5,6], among others.

While model (1) offers reliable performance for both censored and uncensored data due to its ability to incorporate both parametric and nonparametric components, it encompasses only a singular nonparametric component. This constraint necessitates that researchers select a sole nonparametric covariate from the dataset, a premise that might not align with many real-world situations. Furthermore, adhering to this limitation could result in less dependable estimations unless the dataset genuinely contains only one nonparametric covariate. To improve estimation accuracy and provide a more adaptable model that considers the right-censored response variable, $z_{i}$, this research delves into the partially linear additive model (PLAM), tailored for q nonparametric functions:

(3) $$z_{i}=\beta_{0}+x_{i}^{T}\beta +\overset{q}{\underset{j=1}{\sum }}f_{j}\left(t_{ij}\right)+\varepsilon_{i},\;1\leq i\leq n\;$$

Here, q represents the number of nonparametric components, a value determined based on the nature of the relationship between $t_{ij}$ and $y_{i}$. When this relationship cannot be adequately captured by a linear parametric component, it is treated as a nonparametric covariate, characterized by an unknown smooth function $f_{j}\left(t_{ij}\right)$. As a result, the overall nonparametric component of model (3) is formed by the summation of these functions. The use of PLAMs in survival analysis with right-censored data allows for more realistic modeling of the relationship between covariates and survival outcomes by incorporating both multiple parametric and nonparametric components. By introducing nonparametric components, PLAMs provide a more adaptable framework for capturing potential nonparametric relationships between covariates and survival times. It is crucial to acknowledge that model (3) cannot be estimated unless the censorship problem is suitably addressed. Numerous studies in the literature have concentrated on estimating (3) for data that is fully observed and devoid of any censoring. Ref. [7] discussed the combination of smoothing splines with semiparametric additive models, while ref. [8] studied the asymptotic properties of M-estimators for model (3). Additionally, Ref. [9] presented a comprehensive review of partially linear additive models based on various smoothing techniques.

Distinct from the studies previously mentioned, this paper presents modified LLR estimators for PLAM (3) using three distinct censoring solutions: synthetic data transformation (ST), Kaplan–Meier weights (KMW), and kNN imputation (kNNI). Through the examination of these modified estimators and the exploration of various techniques to tackle censorship, valuable insights can be gained, and the accuracy and effectiveness of modeling right-censored data may be improved. This paper also explains the procedure for obtaining these estimators, encompassing the modified backfitting technique and a non-iterative approach, accompanied by comparative numerical studies. To the best of our knowledge, this research fills a gap in the literature on modeling right-censored data.

The remaining part of the paper is organized as follows: In Section 2, the fundamentals of right-censored data are presented, and solution approaches are explained. Section 3 covers the estimation of PLAM using modified LLR estimators based on various censorship solution techniques. In Section 4, the statistical properties of the estimators are provided. Section 5 and Section 6 present simulation and real data studies, respectively. Finally, Section 7 includes the conclusions of the paper.

## 2. Right-Censored Data and Solution Methods

In this section, we provide theoretical insights into modeling right-censored data. Let F and G represent the probability distribution functions of the F observed response variable ($y_{i}$) and the censoring variable ($c_{i}$), respectively. Thus, for any arbitrary data point “u”, these functions can be expressed as follows:

(4) $$F\left(u\right)=P\left(y_{i}\leq u\right)\;\mathrm{and}\;G\left(u\right)=P\left(c_{i}\leq u\right),$$

It is essential to highlight that the estimation procedure for the model, utilizing the specified distributions (4), critically relies on two “censorship assumptions”. These constrain all variables within model (2). These assumptions, as outlined by ref. [10] and elaborated by ref. [11] in the context of right-censored regression models, hold significant significance. In essence, the dataset must meet the subsequent criteria.

**A1.**  $y_{i}$ *and* $c_{i}$ *are independent.*

**A2.**  $P\left(y_{i}\leq c_{i}|y_{i},x_{i},t_{ij}\right)=P\left(y_{i}\leq c_{i}|y_{i}\right)$.

The assumption (A1) and (A2) can be explained as follows: (A2) posits that the covariates in the model lack any information about the censorship in $y_{i}$. Assumption (A1) is particularly crucial when implementing censorship solutions. For a more in-depth discussion, one can refer to [10]’s writings. Drawing from the aforementioned details, this section provides the three censorship solutions. Additionally, towards the section’s close, a figure is showcased to illustrate the practical distinctions between synthetic data transformation and the kNN imputation methods.

Synthetic data transformation: To incorporate the impact of censorship into the modeling procedure, synthetic data transformation is a commonly employed solution method. Consequently, the incomplete response pairs $\left\{\left(z_{i},\;\delta_{i}\right),\;i=1,\ldots ,n\right\}$ must be substituted for a synthetic response variable, as proposed by ref. [12]. Assuming that G is a continuous and known function, it becomes possible to modify the observed lifetimes $z_{i}$ in a manner that ensures an unbiased estimation:

(5) $$z_{iG}=\frac{\delta_{i}z_{i}}{1-G\left(z_{i}\right)},\;i=1,2,\ldots ,n\;$$

where $z_{iG}$ represents the synthetic response variable with $E [z_{iG}|x_{i},t_{ij}]=E [z_{i}|x_{i},t_{ij}]=x_{i}\beta +\sum_{j=1}^{q}f_{j}\left(t_{ij}\right)$. Nevertheless, the true distribution of the censoring variable G remains unknown. To address this challenge, ref. [12] suggested replacing G with its estimated version, known as the Product-Limit estimator (Kaplan–Meier estimator). This estimator calculates the survival probabilities at the arbitrary positive data point “u” as follows:

(6) $$1-\hat{G}\left(u\right)=\prod_{i=1}^{n}{\left(\frac{n-i}{n-i+1}\right)}^{I [z_{\left(i\right)}\leq u,\;\delta_{\left(i\right)}=0]}\;,\;u\geq 0$$

where $z_{\left(1\right)}\leq ,\ldots ,\leq z_{\left(n\right)}$ are the sorted values of the right-censored response variable $z_{\left(i\right)}$ and $\delta_{\left(i\right)}$ are the corresponding censoring indicators associated to $z_{\left(i\right)}$. Hence, instead of $G\left(z_{i}\right)$ in (5), $\hat{G}\left(z_{i}\right)$ is used and $z_{\hat{G}}={\left(z_{1\hat{G}},\ldots ,z_{n\hat{G}}\right)}^{T}$ can be obtained to fit the PLAM. 

Kaplan–Meier weights: Kaplan–Meier weights (KMW), as proposed by ref. [13], are a technique used in survival analysis to address the issue of right-censored data. The Kaplan–Meier estimator is a nonparametric method prevalent nonparametric approach used for estimating survival probabilities amidst censoring. Nonetheless, using standard regression techniques on censored data can lead to biased outcomes. Stute (1993) addressed this by presenting Kaplan–Meier weights, derived from the Kaplan–Meier survival probabilities for each data point. These weights are used to adjust the contribution of each observation in the regression analysis, effectively accounting for the censoring mechanism. By incorporating the Kaplan–Meier weights into the regression model, unbiased estimates of the regression coefficients can be obtained. 

Before computing the KMW, let us assume that $z_{\left(i\right)}$ denotes the ordered values of the incomplete response values and $x_{\left(i\right)}^{T},\;\delta_{\left(i\right)}\;$and $t_{\left(i\right)}=\left(t_{\left(i\right)1},\ldots ,t_{\left(i\right)q}\right)$ are the correspondingly ordered values. Then, Kaplan–Meier weight $w_{\left(i\right)}$, associating with the $z_{\left(i\right)}$, is computed based on the Kaplan–Meier estimator $\hat{F}\left(z_{\left(i\right)}\right)$ given in (5) as follows:

(7) $$w_{\left(i\right)}=\hat{F}\left(z_{\left(i\right)}\right)-\hat{F}\left(z_{\left(i-1\right)}\right)=\frac{\delta_{\left(i\right)}}{n-i+1}\overset{i-1}{\underset{r=1}{\prod }}{\left(\frac{n-r}{n-r+1}\right)}^{\delta_{\left(r\right)}}\;$$

And KMW is obtained for all possible values of $z_{i}$ as a diagonal matrix $W=\mathrm{diag}\left(w_{\left(1\right)},\ldots ,w_{\left(n\right)}\right)$. To reach further information about (7) and implanting these weights into the regression models, see refs. [5,6].

kNN imputation method: kNN imputation is a prevalent technique for addressing missing data across various domains, as discussed by researchers including [14]. Additionally, some studies, such as ref. [15], have adapted the kNN imputation method to manage right-censored data. This method allows for the practical estimation of right-censored data points without the constraints of theoretical limitations. In this context, we provide a succinct overview of the kNN imputation technique and an algorithm tailored for the PLAM dataset. Essentially, the kNN method is a machine learning technique that hinges on the similarity between data points, utilizing distance metrics for predictions. The choice of a suitable similarity measure can greatly impact the results. The Euclidean norm is commonly employed as a measure of distance in numerous studies. The Euclidean norm is a well-known distance and can be computed for the context of censored data points as $d_{E}\left(x_{j},x_{i}\right)=\sqrt{\sum_{i=1}^{n_{c}}{\left(x_{j}^{c}-x_{i}^{c}\right)}^{2}}$ where $n_{c}$ is the number of censored data points and $x_{j}^{c}$ and $x_{i}^{c}$ denote the $j^{th}$ and $i^{th}$ associated values of a regressor which has a strong correlation between response variable $z_{i}$. Details are provided in Algorithm 1. For imputation, the algorithm introduced by ref. [15] can be employed. The choice of the appropriate number of neighbors, “*k*”, is pivotal, especially given the possibility of some neighbors being right-censored. While ref. [16] suggests a smaller value for “*k*”, such as 1 or 2, an optimal “*k*” ranging between 2 to 10 is chosen in this context to minimize the mean squared error (MSE). This approach ensures precision in imputation, taking into account the distinct attributes of the data.
**Algorithm 1** Algorithm for k NN imputation for the right-censored dataInputs$I1:\mathrm{Right}-\mathrm{censored}\;\mathrm{dataset}\;z_{i}$$I2:\mathrm{Censoring}\;\mathrm{indicator}\;\delta_{i}$I3:Number of nearest neigbours k$I4:\mathrm{Values}\;\mathrm{of}\;\mathrm{predictor}\;\mathrm{variable}\;x_{i}\;\;\;\left(\mathrm{high}-\mathrm{correlated}\;\mathrm{one}\;\mathrm{with}\;z_{i}\right)$$Output:\mathrm{Imputed}\;\mathrm{dataset}\;z^{knn}={\left(z_{1}^{knn},\ldots ,\;z_{n}^{knn}\right)}^{T}$^1^: begin^2^: $for\;\left(i=1\;\mathrm{to}\;n\right)$**do**^3^$:\;\;if\left(\delta_{i}=0\right)\;do\;\;\left(\mathrm{if}\;\mathrm{data}\;\mathrm{point}\;\mathrm{is}\;\mathrm{censored}\right)$^4^$:\;\;\;\;\;for\left(j=1\;\mathrm{to}\;n\right)\;$**do**^5^$:\;\;\;\;\;\;\;\;\;\mathrm{Find}\;\mathrm{the}\;\mathrm{distances}\;\mathrm{between}\;x_{j}\;and\;x_{i}\;\mathrm{for}\;\mathrm{each}\;\mathrm{censored}\;\mathrm{data}\;\mathrm{point}$^6^:         Sort the distances from small to large^7^$:\;\;\;\;\;for\;\left(j=1\;\mathrm{to}\;k\right)\;$**do**^8^$:\;\;\;\;\;\;\;\;\;\mathrm{Take}\;\mathrm{the}\;\mathrm{first}\;uncensored\;k\;\mathrm{values}\;\mathrm{of}\;z_{i}\;\mathrm{associated}\;\mathrm{to}\;\mathrm{sorted}\;\mathrm{distances}\;$^9^$:\;\;\;\;\;\;\;\;\;C\mathrm{alculate}\;\mathrm{the}\;i\mathrm{thimputed}\;\mathrm{value}\;\left(z_{i}^{knn}\right)\;\mathrm{with}\;\mathrm{average}\;\mathrm{of}\;\mathrm{nearest}\;k-\mathrm{records}\;\mathrm{of}\;z_{i}$^10^$:\;\;\;\;\;\;\;\;\mathrm{Replace}\;\mathrm{the}\;\mathrm{imputed}\;\mathrm{values}\;\left(z_{i}^{knn}\right)\;\mathrm{with}\;\mathrm{censored}\;\mathrm{data}\;\mathrm{points}\;\left(z_{i},\delta_{i}=0\right)\;\mathrm{in}\;\mathrm{censored}\;\mathrm{data}\;\mathrm{set}\;z=(z_{1},\ldots ,z_{n})$^11^$:\;\mathrm{Return}\;z^{knn}={\left(z_{1}^{knn},\ldots ,\;z_{n}^{knn}\right)}^{T}$^12^: end

**Figure 1:**
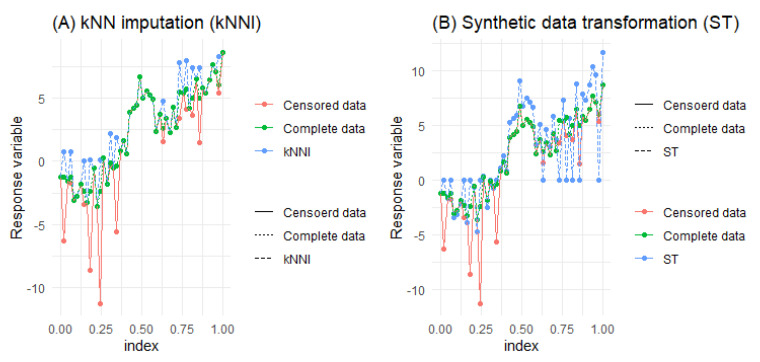
Working procedures of ST in panel (**A**) and KNNI in panel (**B**) for generated data.

As previously mentioned, Figure 1 has been created to illustrate the practical distinctions between the manipulative solution techniques, namely ST and kNNI. This visualization provides insights into how these methods impact the response variable and the changes they bring about. It should be noted that the effect of KMW is not demonstrated in the figure since it is incorporated into the objective function of the right-censored PLAM as weights. However, further explanation regarding KMW will be provided in the next section when obtaining the modified LLR estimators.

## 3. Modified Estimator for PLAM

### 3.1. Fundamentals of PLAM 

Before explaining the modified LLR estimators, this section provides a concise overview of the fundamental concepts of PLAM and summarizes the steps involved in utilizing the backfitting algorithm. Additionally, we express right-censored PLAM (3) in vector and matrix form as follows:

(8) $$Z=\beta_{0}+X\beta +\overset{q}{\underset{j=1}{\sum }}f_{j}+\varepsilon \;$$

Below, we present the explicit expressions for the vector and matrices in (8) as follows:

(9) $$Z= [\begin{array}{c} Z_{1}\\ \vdots \\ Z_{n} \end{array}],\;X= [\begin{array}{c} x_{1}^{T}\\ \vdots \\ x_{n}^{T} \end{array}],\;f_{j}= [\begin{array}{c} f_{j}\left(t_{j1}\right)\\ \vdots \\ f_{k}\left(t_{jn}\right) \end{array}]\;\mathrm{and}\;\varepsilon = [\begin{array}{c} \varepsilon_{1}\\ \vdots \\ \varepsilon_{n} \end{array}]\;$$

The literature offers only a handful of studies specifically addressing the right-censored partially linear additive model (PLAM). In terms of estimating model (8), ref. [17] presented the primary optimization problem for the nonparametric additive model, which mean Xβ=0 in model (8), and ref. [18] formulated a similar problem for (8) as follows:

(10) $$\underset{\beta ,f}{\min}\;E{ [Y-X\beta -\beta_{0}-\overset{q}{\underset{j=1}{\sum }}f_{j}]}^{2}\;$$

Accordingly, the solution expression for the $j^{th}$ function $f_{j}\left(z_{j}\right)$ in the objective (10) can be written as $f_{j}\left(t_{j}\right)=E [\left\{Y-\sum_{k\neq j}f_{k}\left(z_{k}\right)\right\}|\;z_{j}]$ and, based on this statement, the following equation system can be used for the general solution of the model. Accordingly, let $\left(S_{1},\ldots ,S_{q}\right)$ be smoothing matrices obtained from the LLR procedure. Then, the equation system for the estimation of model (8) can be obtained as follows:

(11) $${ [\begin{array}{cccc} I & S_{1} & \cdots & S_{1}\\ S_{2} & I & \cdots & S_{2}\\ \vdots & \vdots & \ddots & \vdots \\ S_{q} & S_{q} & \cdots & I \end{array}]}_{\left(nq\right)\times \left(nq\right)}{ [\begin{array}{c} {\hat{f}}_{1}\\ {\hat{f}}_{2}\\ \vdots \\ {\hat{f}}_{q} \end{array}]}_{\left(nq\times 1\right)}={ [\begin{array}{c} S_{1}\left(Y-X\hat{\beta }\right)\\ S_{2}\left(Y-X\hat{\beta }\right)\\ \vdots \\ S_{q}\left(Y-X\hat{\beta }\right) \end{array}]}_{\left(nq\times 1\right)}$$

where $\hat{\beta }$ denotes estimated coefficients by LLR, which is shown in Section 3.2. For further details on (11), refer to [9]. The solution to system (11) effectively yields the estimates of the functions ${\left\{f_{j}\left(z_{j}\right)\right\}}_{j=1}^{q}$. However, it is evident that inverting the matrix on the left-hand side of (11), which comprises the smoothing matrices, becomes infeasible if the dimension of (nq×nq) is sufficiently large. As the dimension grows, solving the system in (11) becomes progressively more challenging, potentially reaching a point where it is unmanageable and cannot be directly addressed (refer to [18]).

Hence, in practical applications, the system (11) is typically solved using the backfitting method, incorporating initial-valued components notated as ${\left\{{\hat{f}}_{j}^{0}\right\}}_{j=1}^{q}$. Consequently, the LLR estimators are derived by the modified backfitting algorithm, which is given at the end of Section 3.

### 3.2. Local Linear Regression 

Local linear regression (LLR) is a widely employed smoothing technique for nonparametric, semiparametric, and additive models. Its effectiveness has been demonstrated across diverse domains, such as medical research, engineering, and the analysis of time-to-event (or survival) data in time-series studies. In this section, we present three LLR estimators for the partially linear additive model (PLAM) described in (8), employing the introduced censorship solution methods. These estimators are derived using a modified backfitting algorithm. Local linear regression (LLR) is a kernel-based method that differs from kernel regression in that it performs a local estimation of a line rather than a constant. To illustrate the working procedure of LLR, let us consider a partially linear model with a univariate function when q=1, as given in (1), involving an unknown smooth function $f\left(.\right).$ The key concept of LLR is to estimate model (1) linearly within small input intervals. To estimate the parameters of (1), the backfitting algorithm introduced by ref. [19] is used. Accordingly, the backfitting estimators $\left({\hat{\beta }}^{\;},{\hat{f}}^{\;}\right)$ for model (1) where ${\hat{f}}_{1}^{\;}={\left(f_{1}\left(t_{1}\right),\ldots ,f_{1}\left(t_{n}\right)\right)}^{T}$ by replacing the corresponding matrices that are $S_{h_{1}}^{\;}$ and $H_{1}^{\;}$ in the algorithm given in Algorithm 2 can be obtained where $H_{1}^{\;}=S_{h_{1}}^{\;}+\tilde{X}({\left({\tilde{X}}^{\prime }\tilde{X}\right)}^{-1}X^{\prime }\left(I-S_{h_{1}}^{\;}\right)$ for $\tilde{X}=\left(I-S_{h_{1}}^{\;}\right)X$. Here, $S_{h_{1}}^{\;}$ is computed based on the bandwidth parameter $h_{1}>0$ for LLR, which is formed by using nonparametric variables $t_{1i}$’s. 

In order to adapt the LLR method for estimating the parameters of the right-censored PLAM, a closer examination of the elements of the smoother matrix $S_{h_{j}}$ is required. Let ${\left\{S_{h_{j}}^{\;}\right\}}_{j}^{q}$ be written with open form as $S_{h_{j}}^{\;}={\left(s_{j1},\ldots ,s_{jn}\right)}^{T}$, where $\left(s_{j1},\ldots ,s_{jn}\right)$ show the row vectors of $S_{h_{j}}^{\;}$ obtained from values of $h^{th}$ nonparametric covariate $t_{j}={\left(t_{j1},\ldots ,z_{jn}\right)}^{T}$. From the theory of LLR, $s_{jr}^{T}$ for any $t_{j1}\leq m\;\leq t_{jn}\;$can be obtained as follows:

$$s_{jm}^{T}=d_{1}^{T}{\left(t_{jm}^{T}W_{jm}t_{jm}\right)}^{-1}t_{jm}^{T}W_{jm}$$

where $t_{jm}$, $d_{1}$, and $W_{jm}$ can be expressed as follows:

$$t_{jm}= [\begin{array}{cc} 1 & \left(t_{j1}-m\right)\\ \vdots & \vdots \\ 1 & \left(t_{jn}-m\right) \end{array}],\;d_{1}= [\begin{array}{c} 1\\ 0 \end{array}]\;$$

and

(12) $$W_{jm}=\mathrm{diag} [h^{-1}K\left(\frac{t_{j1}-m}{h}\right),\cdots ,h^{-1}K\left(\frac{t_{jn}-m}{h}\right)]\;$$

Based on the provided information, it can be inferred that the extension of LLR estimators to PLAM requires further adjustments. Moreover, it is crucial to satisfy the standard assumptions of LLR, such as where $K\left(.\right)$ is the kernel function, which is continuous, and its moment is written as $\mu_{i}\left(K\right)\equiv \int u^{i}K\left(u\right)du=0$ when $\mu_{2}\left(K\right)\neq 0$ for odd values of j. The density of $t_{ji}$ can be given as $g_{t}\left(m\right)>0$, for all $m\in sup\left(g_{t}\right)$, and also, as a common assumption, since n→∞, h→0, and nh→∞. Finally, a second derivative of the nonparametric smooth function $f\left(.\right)$ exists and is continuous. Details about the assumptions are discussed in detail in ref. [20].

In the backfitting estimation procedure, to make simple the definition of the model (8), some restrictions on ${\left\{f_{j}\left(t_{ij}\right)\right\}}_{j=1}^{q}$ are needed. At first, $E [f_{j}\left(t_{ij}\right)]=0$ is assumed. Secondly, the parametric covariates $x_{i}^{T}$’s and right-censored response values $z_{i}$’s are assumed to be scaled around zero. In order to construct the centered smoother matrix $S_{h_{j}}$ used in the LLR estimation, these constraints are necessary. Thus, the conditional expectation of model (8) can be expressed as follows:

(13) $$E\left(z_{i}|x_{i},t_{i}\right)=\beta_{0}+x_{i}^{T}\beta +\overset{q}{\underset{j=1}{\sum }}f_{j}\left(t_{ij}\right),\;i=1,\ldots ,n\;$$

By using the modified backfitting algorithm given in Algorithm 2, solutions can be obtained based on $S_{h_{j}}^{\;}$ for PLAM parameters β and ${\left\{f_{j}\right\}}_{j=1}^{q}$. Thus, without any censoring adjustment, PLAM estimators $\left({\hat{\beta }}^{\;},{\hat{f}}^{\;}\right)$ based on the LLR are obtained.
**Algorithm 2** Modified Backfitting Algorithm for Right-Censored PLAM**Inputs**: $\;\;\;\beta_{0}=E\left(Z_{i}\right)=\bar{Z}$;  X: $\left(n\times p\right)$-dimensional covariates of parametric component
$\;\;\;\;\;\;\;\;\;\;\;\;Z:\left(n\times q\right)$-dimensional scaled nonparametric covariates; ${\left\{f_{k}^{\left(0\right)}\right\}}_{k=1}^{q}$: Initial smooth functions
$\;\;\;\;\;\;\;\;\;\;\;\;\beta^{\left(0\right)}:\;$Initial regression coefficients; $Z^{\;}$: $\left(n\times 1\right)$-dim. vector of right-censored response values
                   Tolerance value, tol=0.05 and max. iteration = 100.
**Outputs**: Modified PLAM estimators:
                    **O1**:kNNI basis LLR estimators ${\hat{\beta }}^{imp}\;\mathrm{and}\;\left({\hat{f}}_{1}^{imp},\ldots ,{\hat{f}}_{q}^{imp}\right)$
                    **O2**:ST basis estimators ${\hat{\beta }}^{ST}\;\mathrm{and}\;\left({\hat{f}}_{1}^{ST},\ldots ,{\hat{f}}_{q}^{ST}\right)$
                    **O3**: KMW basis estimators ${\hat{\beta }}^{KMW}\;\mathrm{and}\;\left({\hat{f}}_{1}^{KMW},\ldots ,{\hat{f}}_{q}^{KMW}\right)$
**Begin**^1:^ Initialize β and $\left(f_{1},\ldots ,f_{q}\right)$ as $\beta^{\left(0\right)}$ and ${\left\{f_{j}^{\left(0\right)}\right\}}_{j=1}^{q}$ by covariates X and $t_{1},\ldots ,t_{q}$.
^2:^ **while** $\left(tol\geq 0.05\right)$ and $\left(i<max.iteration\right)$
*Selection of optimal bandwidth parameter* $h_{j}$ *by* GCV *between steps: 3–8*^3:^   Create a sequence of tunning parameter $h_{seq}= [0.01,\;1.5]$ for determined length
^4:^          **for** (l in 1:length) **do**
^5:^                Compute the smoothing matrix $S_{h_{seq}}^{\left(l\right)}$.
^6:^                 **if** censorship solution is **KMW**
^7:^                  Compute $\tilde{X}$ and $H_{j}^{\left(l\right)}=S_{h_{seq}}^{\left(l\right)}+\tilde{X}({\left({\tilde{X}}^{\prime }W\tilde{X}\right)}^{-1}X^{T}W\left(I-S_{h_{seq}}^{\left(l\right)}\right)$ where $\tilde{X}=\left(I-S_{h_{seq}}^{\left(l\right)}\right)X$
^8:^                 **Else**^9:^                  Compute $\tilde{X}$ and $H_{j}^{\left(l\right)}=S_{h_{seq}}^{\left(l\right)}+\tilde{X}({\left({\tilde{X}}^{\prime }W\tilde{X}\right)}^{-1}X^{T}W\left(I-S_{h_{seq}}^{\left(l\right)}\right)$ where $\tilde{X}=\left(I-S_{h_{seq}}^{\left(l\right)}\right)X$
^10:^                Calculate GCV$\left(h_{seq}^{\left(l\right)}\right)$ as given in Equation (24)
^11:^          **end**^12:^          Select optimal ${\hat{h}}_{j}$ which minimizes $GCV\left(h_{j}\right)$ for $j^{th}$ function $f_{j}$.
^13:^          Compute $S_{{\hat{h}}_{j}}$ for each criterion (and method).
                                               *Solution of censorship problem between steps: 14–25*
^14:^          **if** the censorship solution is **kNNI**
^15:^                  Replace Z with $Z^{imp}$ using algorithm in Algorithm 1.
^16:^          **if** the censorship solution is **ST**
^17:^                  Replace Z with $Z^{ST}$ as shown in Equation (5)
^18:^          **for** $\left(j\;in\;1:q\right)$ **do**^19:^                  **if** the censorship solution is **KMW**
^20:^                                 ${\hat{\beta }}_{j}^{\left(i\right)}={\left(X^{\prime }WX\right)}^{-1}X^{\prime }W\left(Z-\beta_{0}-\sum_{m<j}^{q}{\hat{f}}_{m}^{\left(i\right)}-\sum_{m>j}^{q}{\hat{f}}_{m}^{\left(i-1\right)}\right)\;$^21:^                                 ${\hat{f}}_{j}^{\left(i\right)}=S_{{\hat{h}}_{j}}\left(Z-\beta_{0}-X{\hat{\beta }}_{j}^{\left(i\right)}-\sum_{m<j}^{q}{\hat{f}}_{m}^{\left(i\right)}-\sum_{m>j}^{q}{\hat{f}}_{m}^{\left(i-1\right)}\right)$^22:^                  **Else**^23:^                                 ${\hat{\beta }}_{j}^{\left(i\right)}={\left(X^{\prime }X\right)}^{-1}X^{\prime }\left(Z-\beta_{0}-\sum_{m<j}^{q}{\hat{f}}_{m}^{\left(i\right)}-\sum_{m>k}^{q}{\hat{f}}_{m}^{\left(i-1\right)}\right)\;$^24:^                                 ${\hat{f}}_{j}^{\left(i\right)}=S_{{\hat{\lambda }}_{k}}\left(Y-\alpha_{0}-X{\hat{\beta }}_{k}^{\left(i\right)}-\sum_{m<k}^{q}{\hat{f}}_{m}^{\left(i\right)}-\sum_{m>k}^{q}{\hat{f}}_{m}^{\left(i-1\right)}\right)$^25:^          **end**^26:^                  i=i+1^27:^                  $tol={\left(nq\right)}^{-1}\left|{\left(f_{k}^{\left(i\right)}-f_{k}^{\left(i-1\right)}\right)}^{T}1\right|\;\mathrm{where}\;1={\left(1,\ldots ,1\right)}^{T}.$^28:^  **end**^29:^ **Return** $\hat{\beta }\;\mathrm{and}\;\left({\hat{f}}_{1},\ldots ,{\hat{f}}_{q}\right)$^30:^ **end**

Furthermore, it should be noted that ref. [20] presented a non-iterative formulation equivalent to the backfitting algorithm based on an additive smoother matrix $S_{\;}^{A}=\sum_{j=1}^{q}S_{j}^{\;*}$ to demonstrate the LLR estimation process in the absence of censorship issues, which reveals the relationship between Z and ${\hat{f}}_{\;}^{A}=\sum_{j=1}^{q}{\hat{f}}_{j}^{\;}$. Here, $S_{j}^{*}$ is computed from the equation system (11) based on the $S_{h_{j}}^{\;}$ (see ref. [9]). Additionally, this information elucidates the connection between a unique solution and the iterative backfitting process.

Accordingly, LLR estimators for PLAM can be found as for both ST and kNNI by replacing Z by $Z^{ST}$ and $Z^{kNNI}$:

(14) $${\hat{\beta }}^{A}={\left(X^{T}\tilde{X}\right)}^{-1}X^{T}\tilde{Z}$$

(15) $${\hat{f}}_{\;}^{A}=S_{\;}^{A}\left(Z-\alpha_{0}-X{\hat{\beta }}^{A}\right)$$

And for KMW solution, non-iterative estimators are obtained as follows:

(16) $${\hat{\beta }}_{KMW}^{A}={\left(X^{T}W\tilde{X}\right)}^{-1}X^{T}W\tilde{Z}$$

(17) $${\hat{f}}_{KMW}^{A}=S_{\;}^{A}\left(Z-\alpha_{0}-X{\hat{\beta }}^{A}\right)$$

where $\tilde{X}=\left(I-S_{\;}^{A}\right)X$, $\tilde{Z}=\left(I-S_{\;}^{A}\right)Z$. It should be noted that the validity of Equations (14)–(17) depends on the existence of a unique solution. Furthermore, the vector of fitted values for LLR can be expressed as follows:

(18) $${\hat{\mu }}_{\;}=E [Z|X,Z]={\hat{Z}}_{\;}=H_{\;}^{A}Z\;$$

where $H_{\;}^{A}=S_{\;}^{A}+\tilde{X}{ [{\tilde{X}}^{T}\tilde{X}]}^{-1}X^{T}\left(I-S_{\;}^{A}\right)$ and for the KMW solution $H_{KMW}^{A}=S_{\;}^{A}+\tilde{X}{ [{\tilde{X}}^{T}W\tilde{X}]}^{-1}X^{T}W\left(I-S_{\;}^{A}\right)$. Note that under completely observed data, $H_{\;}^{A}$ is derived by [21] for the LLR estimator of PLAM. 

To effectively demonstrate and interpret each nonparametric component individually, the introduced modified backfitting algorithm is more suitable than Equations (16)–(18), which yield an additive outcome for the nonparametric component. Additionally, computing $S_{LL}^{A}$ becomes significantly challenging as the dimension of the additive component increases. In this paper, the modified backfitting estimators $\left({\hat{\beta }}^{A},{\hat{f}}^{A}\right)$ of LLR, obtained through an algorithm given in Algorithm 2, are employed. This approach aims to showcase the performance of the estimated functions $\hat{f}={\left\{{\hat{f}}_{j}^{\;}\right\}}_{j=1}^{q}$. In the introduced algorithm given in Algorithm 2, to calculate the selection criterion GCV, the degrees of freedom of (DF) are computed by $DF_{j}=tr\lceil {\left(I-H_{j}\right)}^{T}\left(I-H_{j}\right)\rceil =n-2tr\left(H_{j}\right)+tr\left(H_{j}^{T}H_{j}\right)$ where $H_{j}$ denotes the hat matrix based on the $j^{th}$ nonparametric component. Also, to see details about the algorithm given in Algorithm 2, see ref. [9].

## 4. Properties of the Estimator 

The objective of this section is to assess the bias and variance of the modified LLR estimators introduced in the previous section. When evaluating the performance of the parametric component, the variances and biases of the regression coefficients are calculated using the non-iterative solutions given in Equations (14)–(17), owing to its theoretical simplicity. 

Empirical studies can be conducted to calculate the bias and variance properties of the estimators. However, when considering LLR as demonstrated in Equations (14)–(17), non-iterative formulations can be employed to compute finite-sample properties for the other two methods. In this matter, conditional bias $E [\left({\hat{\beta }}^{A}-\beta \right)|X,t]$ and variance $Var\left({\hat{\beta }}^{A}\right)$ are obtained based on Equations (14)–(17).

Let us rewrite ${\hat{\beta }}^{A}$ as:

$${\hat{\beta }}^{A}=\beta +{\left(X^{T}\tilde{X}\right)}^{-1}X^{T}{\tilde{f}}_{\;}^{A}+{\left(X^{T}\tilde{X}\right)}^{-1}X^{T}\left(I-S_{\;}^{A}\;\right)\varepsilon$$

where $S_{\;}^{A}=\sum_{j=1}^{q}S_{j}^{*},$ and ${\tilde{f}}_{\;}^{A}=\left({\tilde{f}}_{1}+\ldots +{\tilde{f}}_{q}\right)$ for ${\left\{{\tilde{f}}_{j}=\left(I-S_{h_{k}}^{\;}\right)f_{j}\right\}}_{j=1}^{q}$**.** Then $B\left({\hat{\beta }}^{A}\right)$ and $Var\left({\hat{\beta }}^{A}\right)$ can be given by:

(19) $$B\left({\hat{\beta }}^{A}\right)=E [\left({\hat{\beta }}^{A}-\beta \right)|X,t]={\left(X^{T}\tilde{X}\right)}^{-1}X^{T}{\tilde{f}}_{\;}^{A}$$

(20) $$Var\left({\hat{\beta }}^{A}\right)={\hat{\sigma }}_{\varepsilon }^{2}{\left(X^{T}\tilde{X}\right)}^{-1}X^{T}{\left(I-S_{\;}^{A}\;\right)}^{2}X{\left(X^{T}\tilde{X}\right)}^{-1}$$

And for the KMW solution, Equations (19) and (20) are given by:

(21) $$B\left({\hat{\beta }}_{KMW}^{A}\right)=E [\left({\hat{\beta }}^{A}-\beta \right)|X,t]={\left(X^{T}W\tilde{X}\right)}^{-1}X^{T}W{\tilde{f}}_{KMW}^{A}$$

(22) $$Var\left({\hat{\beta }}_{KMW}^{A}\right)={\hat{\sigma }}_{\varepsilon }^{2}{\left(X^{T}W\tilde{X}\right)}^{-1}X^{T}W{\left(I-S_{\;}^{A}\;\right)}^{2}X{\left(X^{T}W\tilde{X}\right)}^{-1}$$

where ${\hat{\sigma }}_{\varepsilon }^{2}$ is the model variance estimated based on LLR and it can be computed using the hat matrix $H_{\;}^{A}$ or $H_{KMW}^{A}$ for the KMW solution that are defined after Equation (18). In addition, one can replace Z by $Z^{ST}$ or $Z^{imp}$. Accordingly, ${\hat{\sigma }}_{\varepsilon }^{2}$ is formulated as follows:

(23) $${\hat{\sigma }}_{\varepsilon }^{2}=\frac{Z^{T}{\left(I-H_{\;}^{A}\right)}^{T}\left(I-H_{\;}^{A}\right)Z}{tr [{\left(I-H_{LL}^{A}\right)}^{T}\left(I-H_{LL}^{A}\right)]}$$

where the degree of freedom (DF), which is given in the denominator of (23), is calculated by $DF_{A}=tr [{\left(I-H_{\;}^{A}\right)}^{T}\left(I-H_{\;}^{A}\right)]=n-2tr\left(H^{A}\right)+tr\left({\left(H^{A}\right)}^{T}\;H^{A}\right)$ and $H_{KMW}^{A}$ is used for the KMW solution. For the further details of $DF_{A}$, see ref. [17]. The modified backfitting algorithm provided in Algorithm 2 requires the estimation of the model variance for each individual nonparametric function in order to calculate the GCV score for bandwidth parameter selection. Consequently, if $H_{\;}^{A}$ is replaced by $H_{j}$ or $H_{KMW_{j}}$ in (23), then the individual variance estimator ${\hat{\sigma }}_{\varepsilon_{j}}^{2}$ can be easily obtained. The fundamental concept behind computing ${\hat{\sigma }}_{\varepsilon_{j}}^{2}$ lies in selecting the appropriate smoothing and bandwidth parameters using the GCV criterion, as it relies on the estimated model variance. The GCV criterion can be summarized as follows.

GCV**criterion**: Generalized cross-validation is used to obtain a minimum score based on the optimal tuning parameter for the regression model. In terms of bandwidth selection in additive models with LLR, ref. [22] presented a detailed work on using GCV and its properties. Accordingly, to choose the optimal $h_{j}$ for $j^{th}$ function $f_{j}$, $GCV\left(h_{j}\right)$ score can be computed based on ${\hat{\mu }}_{\;}$ given in (18):

(24) $$GCV\left(h_{j}\right)=\frac{{\left(Z-\hat{\mu }\right)}^{T}\left(Z-\hat{\mu }\right)}{n{\left\{1-\left(n^{-1}tr\left(H_{j}\right)\right)\right\}}^{2}\;}\;$$

where $H_{j}$ is the hat matrix obtained for $f_{j}$ which is provided at the end of the Section 3. Notice that calculating the true $DF_{j}$ in PLAM is asymptotically justifiable if parametric and nonparametric covariates $\left(x_{i},t_{j}\right)$ are independent. If there is multicollinearity, then Equation (24) may be regularized properly due to overestimated $DF_{j}$.

### 4.1. Evaluation of Performance

#### 4.1.1. Metrics for the Parametric Component

In this section, two metrics are presented to assess the performance of the LLR estimator of the parametric component of the model $\hat{\beta }$ that are scalar versions of the dispersion error (SMDE) and the relative efficiency (RE), which is computed by ratio of the SMDE values. The formulations are given below:

(25) $$SMDE\left({\hat{\beta }}_{\;},\beta \right)=E\lceil {\left(\beta -\hat{\beta }\right)}^{\prime }\left(\beta -\hat{\beta }\right)\rceil =tr [\left(MSE\left(\hat{\beta },\beta \right)\right)]$$

where $MSE\left(\hat{\beta },\beta \right)$ is expressed as a summation of bias square and variance of $\hat{\beta },$ and given by:

(26) $$MSE\left(\hat{\beta },\beta \right)=E\lceil {\left(\beta -\hat{\beta }\right)}^{\prime }\left(\beta -\hat{\beta }\right)\rceil =Var\left(\hat{\beta }\right)+{ [B\left(\hat{\beta }\right)]}^{2}$$

Then, using (25), REs of the methods on estimating β can be computed. In this paper, methods are considered for use as censorship solution techniques for REs. 

Let ${\hat{\beta }}_{1}$ and ${\hat{\beta }}_{2}$ represent the estimates of parametric components based on two different censorship solutions. Accordingly, RE can be formulated as follows:

(27) $$RE\left({\hat{\beta }}_{1},{\hat{\beta }}_{2}\right)=SMDE\left({\hat{\beta }}_{1},\beta \right)/SMDE\left({\hat{\beta }}_{2},\beta \right)\;\;$$

where $RE\left({\hat{\beta }}_{1},{\hat{\beta }}_{2}\right)<1$ indicates that ${\hat{\beta }}_{1}$ is more efficient than ${\hat{\beta }}_{2}$.

#### 4.1.2. Metrics for the Nonparametric Component

To evaluate the quality of the estimated nonparametric component, two measures are presented. The first measure is the root mean squared error (RMSE), which measures the accuracy of each individual estimated function in the model. The second measure is the averaged root mean squared error (ARMSE) which is specifically designed to assess the performance of the overall additive component $\hat{f}=\left({\hat{f}}_{1},\;\ldots ,{\hat{f}}_{q}\right)$. The formulations of RMSE and ARMSE are written as:

(28) $$RMSE_{j}\left(f_{j},{\hat{f}}_{j}\right)=\sqrt{n^{-1}\overset{n}{\underset{i=1}{\sum }}{ [f_{j}\left(z_{ij}\right)-{\hat{f}}_{j}\left(z_{ij}\right)]}^{2}},\;1\leq j\leq q\;$$

and

(29) $$ARMSE\left(f^{A},{\hat{f}}^{A}\right)=q^{-1}\overset{q}{\underset{j=1}{\sum }}RMSE_{j}\left(f_{j},{\hat{f}}_{j}\right)\;$$

where $f=\sum_{j=1}^{q}f_{j}$ and $\hat{f}=\sum_{j=1}^{q}{\hat{f}}_{j}$. 

## 5. Simulation Study

The practical performance of the modified LLR estimators in the context of right-censored PLAM with various censorship solution methods is analyzed in this section. To achieve this, different settings for sample size (n), the number of additive nonparametric components (q), and the level of censoring (CL) are considered. Specifically, three sample sizes (n=50, 100, and 200) and three levels of censoring (CL=5%, 20%, and 35%) are chosen. A total of eight scenarios are obtained by combining these configurations. Additionally, a total of 24 cases for analysis are formed by using three censorship solution methods. Moreover, accelerated failure time model estimation results are presented as benchmark performance scores. To achieve that existing function, the survival library in R is used. Note that the function written in R for this paper is provided via link: https://github.com/yilmazersin13/Censored-Partially-linear-additive-models/tree/main, accessed on 9 August 2023. The simulation design and setup used in this study are designed in a manner commonly found in the literature (see ref. [4]). Small, medium, and large sample sizes are chosen, along with three different censoring levels, in accordance with reference articles. Furthermore, the nonparametric component count has been determined in two distinct ways, introducing a novel approach that differs from most similar studies (see ref. [9]). 

After establishing the design, the data generation procedure for the right-censored PLAM is outlined here. Firstly, PLAM with completely observed responses is generated as:

(30) $$y_{i}=x_{i}^{T}\beta +\overset{q}{\underset{j=1}{\sum }}f_{j}\left(t_{ji}\right)+\varepsilon_{i},\;1\leq i\leq n\;$$

where $x_{i}^{T}={\left(x_{i1},x_{i2}\right)}^{T}$, is $\left(n\times 2\right)$ dimensional parametric covariate matrix with normally distributed and independently $x_{i}$’s that are generated as $x_{i}\sim N\left(\mu_{x}=0,\sigma_{x}^{2}=1\right)$. Also, the vector of regression coefficients is determined as $\beta ={\left(1,-0.5\right)}^{T}$**.** Regarding the nonparametric component, smooth functions are generated by $f_{1}\left(t_{1}\right)=1-48t_{1}+218t_{1}^{2}-315t_{1}^{3}+145t_{1}^{4}$ with $t_{1}={\left\{\left(i-0.5\right)/n\right\}}_{i=1}^{n}$ and $f_{2}\left(t_{2}\right)=\sin\left(2t_{2}\right)+2e^{-16t_{2}^{2}}$ with $t_{2}=U [-2,\;2]$ when q=2. Note that, due to how all the variables are scaled in the simulation study, the constant term $\alpha_{0}$ is not used throughout the section. Finally, the random error terms $\varepsilon_{i}$’s are independent and identically distributed with zero mean and constant variance, which can be shown as $\varepsilon_{i}\sim N\left(0,\sigma_{\varepsilon }^{2}=0.5\right)$. 

After generating (30), by applying the censorship procedure given in Algorithm 3, right-censored response variable Z is generated based on random censoring variable $C={\left(c_{1},\ldots ,c_{n}\right)}^{T}\;$and censoring indicator $\delta ={\left(\delta_{1},\ldots ,\delta_{n}\right)}^{T}$.
**Algorithm 3** Censoring Procedure**Input:** Completely observed $y_{i}$
**Output:** Right-censored dependent variable $z_{i}$
^1^: For given censoring level (CL), produce $\delta_{i}=I\left(y_{i}\leq c_{i}\right)$ from the binomial distribution
^2^: **for** $\left(i\;in\;1\;to\;n\right)$^3^:             **If** $\left(\delta_{i}=0\right)$^4^:                     **while** $\left(y_{i}\leq c_{i}\right)$^5^:                     generate $c_{i}\sim N\left(\mu_{y},\sigma_{y}^{2}\right)$^6^:             **Else**^7^:                      $c_{i}=z_{i}$^8^: **end** (for loop in Step 2)
^9^: **for** $\left(i\;in\;1\;to\;n\right)$^10^:          **If** $\left(y_{i}\leq c_{i}\right)$^11^:                     $z_{i}=y_{i}$^12^:          **Else**^13^:                     $z_{i}=c_{i}$^14^: **end** (for loop in Step 9)

Then, right-censored PLAM is obtained with the incomplete response variable $Z={\left(Z_{1},\ldots ,Z_{n}\right)}^{T}$. Accordingly, the following figures and tables are provided based on the censorship solution techniques. Algorithms 2 and 3 present the results for the performance of the parametric component estimation, specifically the SMDE and RE values, respectively. In addition, as a benchmark method, the performance of AFT model estimation based on Cox’s semiparametric proportional hazards (CPH) estimator is provided in both simulation and real data examples. The estimates are obtained a using “*Survival*” package in R.

**Figure 2:**
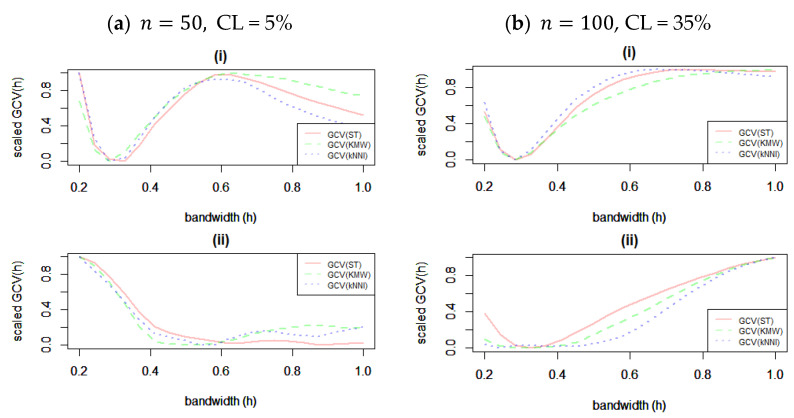
Selection of bandwidth parameter (h) for different scenarios and censorship solution methods when n=50. In each panel, (i) and (ii) involve the selection processes for $f_{1}\left(t_{1}\right)$ and $f_{2}\left(t_{2}\right)$, respectively.

Prior to presenting the findings, we offer a visual representation in Figure 2 that elucidates the process of bandwidth selection across diverse scenarios. This illustration sheds light on how the choice of bandwidth is intricately intertwined with the extent of censoring and the specific methods employed for addressing censorship. The discerning eye will note that in the context of $f_{1}$, the selection of bandwidth appears to exhibit a lesser degree of sensitivity to variations in the level of censoring and sample size. However, in the case of the $f_{2}\;$function, it becomes clear that the level of censorship exerts a discernible influence on the chosen bandwidth value. Notably, when confronted with elevated censorship levels across all solution strategies, a preference for smaller bandwidths becomes evident. This outcome is intuitively reasonable since, especially in scenarios involving ST and kNNI, the structural complexity of the data to be fitted takes on a more undulating nature. Therefore, it is evident that we can extrapolate that accounting for the degree of censorship is a pivotal factor when navigating the terrain of bandwidth selection. These findings resonate with prior research in this domain. Ref. [23] demonstrated similar behavior in a related context, highlighting the sensitivity of bandwidth to censorship levels. In line with the in-depth investigations of ref. [24], our observations underscore the need for cautious bandwidth selection in scenarios characterized by substantial censorship, promoting the accurate modeling of intricate data structures.

The results in Table 1 demonstrate that the estimation quality of the modified LLR estimators for the parametric component β improves with lower censoring levels and larger sample sizes across all censorship techniques. These tendencies align with the expected theoretical behavior. Specifically, the LLR-KMW estimator exhibits dominant performance in many simulation combinations, closely followed by the LLR-kNNI estimator with competitive SMDE scores. However, the LLR-ST does not yield good performance. Also, as a benchmark method for the model, SMDE scores of the CPH estimator are presented in the table. It is evident that due to the model involving serious complexity with two different nonparametric functions, there is a significant distance between the LLR-based estimators and the CPH estimator, which is expected. 

Interestingly, in cases where n=50 and CL=5% or CL=20%, the LLR-kNNI estimator outperforms the LLR-KMW estimator. As the sample size increases, LLR-KMW takes the lead, in accordance with its theoretical behavior. It is worth noting that due to its fully nonparametric nature, LLR-kNNI may yield better results under different configurations, demonstrating relative independence from specific simulation settings. This characteristic is observed in the combination of n=200 and CL=20%.

Additionally, to assess the impact of censorship on the solution techniques, the increase in SMDE scores between censorship levels is examined. The results indicate that the the LLR-ST estimator is the most affected by censorship, which aligns with the theoretical background of ST presented in Section 2.

In Table 2, the calculation of the RE scores follows a decision where the nominators represent the columns, and the denominators represent the rows. Therefore, an RE value of less than 1 in Table 2 indicates that the method in the column is more effective than the methods in the corresponding row. Please note that, for the sake of saving space, only certain simulation configurations are considered in Table 2. The results in the table confirm that LLR-KMW is more efficient than LLR-ST in all cases. Simultaneously, LLR-KMW and LLR-kNNI exhibit similar outcomes, indicating that they are not distinctly efficient in any simulation configurations for estimating the parametric component of the PLAM. 

Furthermore, when the censoring level is very high (CL=35%), the RE scores deviate from 1, making the performance differences among the LLR estimators based on the solution techniques more apparent. Once again, it is evident that, especially for n=50, ST is the most sensitive technique to censorship compared with the other two methods. Additionally, the results reveal that LLR-kNNI and LLR-KMW display similar RE scores in every combination. In addition, in Table 2, REs of CPH show that there is a clear dominance of LLR-basis estimators for the estimation of right-censored PLAM. This result also proves that the introduced estimator has important potential to be an alternative estimator for the model of interest that is used in survival analysis.

**Figure 3:**
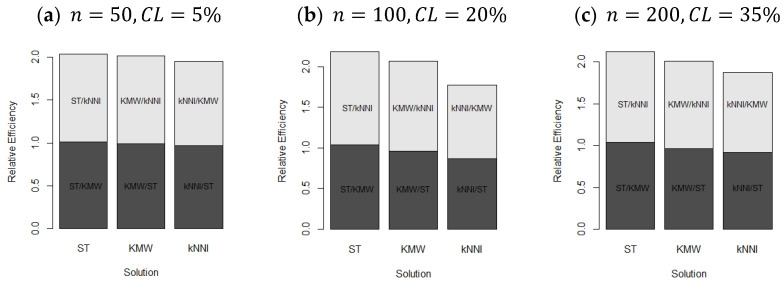
Bar plots of averaged RE scores.

In Figure 3, the averaged values of the RE scores are displayed, confirming the interpretations from Table 2. The figure also shows both the effects of censorship and the sample size. In panel (a), the RE values are very close to each other due to the very low censoring level (CL=5%). Panels (b) and (c) demonstrate the change in RE scores as the censoring level increases, with the differences between the estimators becoming more distinct, as mentioned earlier. Consequently, the LLR-kNNI and LLR-KMW estimators are more efficient than the LLR-ST estimator. In panel (c), the performances are once again close to each other, reflecting the large sample size (n=200).

After analyzing the parametric component, the estimation of the additive nonparametric components is presented in Table 3 and Table 4. Table 3 displays the RMSE values computed for the individual functions, while Table 4 provides the ARMSE values for all simulation configurations, serving as a measure of the overall performance in estimating the nonparametric component of the right-censored PLAM. Upon initial examination, the LLR-KMW estimator demonstrates a significantly superior performance compared with the other two estimators across all simulation configurations. This dominance is further evidenced by the ARMSE results presented in Table 4, which contrast the outcomes observed in the parametric component estimation. 

An interesting distinction in estimating the nonparametric component is that the performances of the introduced estimators deteriorate as the sample size increases. To explain this phenomenon, it is crucial to note that in the estimation of PLAMs, there exists a balance between the estimation of parametric and nonparametric components, which exhibits an inverse relationship. Furthermore, when data points are scattered widely around the representative smooth curve, the bias of the fitted curve increases. Additionally, the RMSE scores for the three modified LLR estimators are fairly similar to each other, confirming that the modified backfitting algorithm functions effectively with the censorship solution techniques. 

**Figure 4:**
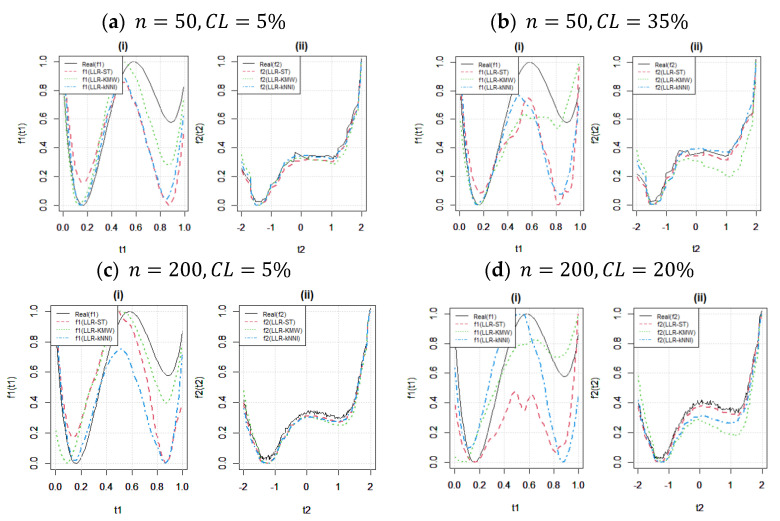
Fitted curves to show the effect of the censoring level (CL). In each panel, (i) and (ii) show fitted curves for $f_{1}\left(t_{1}\right)$ and $f_{3}\left(t_{2}\right)$ respectively.

**Figure 5:**
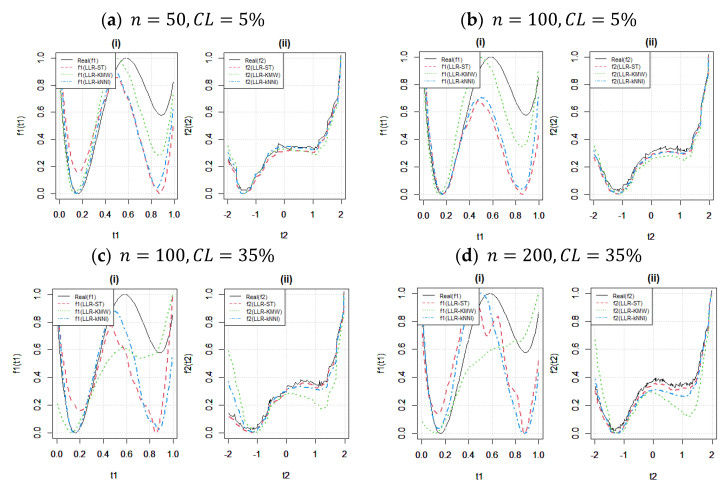
Fitted curves to show the effect of the sample size (n). In each panel, (i) and (ii) show fitted curves for $f_{1}\left(t_{1}\right)$ and $f_{3}\left(t_{2}\right)$ respectively.

Table 4 presents a strong case, confirming the dominant role of the LLR-KMW estimator in estimating nonparametric components within the context of right-censored PLAM. The success of the LLR-KMW estimator lies in its clever use of weighted estimation, which works well for both the parametric and nonparametric aspects of PLAM. Notably, the LLR-KMW estimator does not just improve **β** estimates, it also works well together with the LLR-kNNI estimator, forming a powerful estimation duo. When we carefully analyze Table 4 and take a close look at Figure 4 and Figure 5, a clear pattern emerges. Both the LLR-KMW and LLR-kNNI estimators perform very similarly when it comes to estimating the nonparametric component. What is even more interesting is that both estimators outperform the LLR-ST estimator, as these enlightening visuals below beautifully demonstrate. In terms of estimating nonparametric components, it is naturally expected that the CPH estimator does not show a good performance due to its theoretical structure. However, its behaviors are similar to LLR-basis estimators in sample size and censoring level changes. In summary, the introduced LLR-basis estimators show better performance than the classical CPH estimator. 

Figure 4 illustrates the behavior of the estimators under different censoring levels with fixed sample sizes. In panels (a)–(b), the effect of the censoring level is investigated when the sample size is small (n=50). It can be observed that while $f_{2}\left(t_{2}\right)$ is not significantly affected, the estimate of $f_{1}\left(t_{1}\right)$ is heavily influenced by the censored data points. It is important to note that this inference is also related to the initial values $\left(\beta^{\left(0\right)},f^{\left(0\right)}\right)$ determined in the algorithm and their compatibility with the unknown functions $f_{1}$ and $f_{2}$, respectively (see [9] for further discussions). Furthermore, the results demonstrate that the weakness of the LLR-ST estimator (red dotted line) is clear in all four panels (a), (b), (c), and (d), for both n=50 and n=200. Additionally, panels (c) and (d) support the findings of Table 3 and Table 4, leading to the conclusion that, for larger sample sizes, the fitted curves become more sensitive to the censoring level, resulting in a decrease in their performance.

Figure 5 investigates the effect of sample size (n) for fixed censoring levels in the upper and lower panels, particularly for CL=35% in panels (c) and (d), while LLR-KMW and LLR-ST exhibit a slightly more pronounced response to increasing sample size compared with LLR-kNNI. This result is expected due to the nonparametric nature of kNNI. Furthermore, the changes observed in the fitted curves are more noticeable for the estimation of $f_{1}\left(t_{1}\right)$, as shown in Figure 4. Additionally, the differences between sample sizes for the lower censoring level (CL=5%) in panels (a)–(b) indicate that there is minimal variation between the fitted curves for both functions.

These trends are consistent with the findings reported by ref. [25], where a similar sensitivity of the ST basis estimator to sample size was identified in a related context. The reaction of the kNNI, KMW, and ST estimators to sample size fluctuations aligns with the observations made by ref. [26] reinforcing the notion that these estimators can exhibit greater flexibility in accommodating varying sample sizes. 

To assess the performance of the introduced modified LLR estimators on real-world data and compare them with the simulation results, a real data example is presented in the following section, focusing on the hepatocellular carcinoma dataset.

## 6. Hepatocellular Carcinoma Data Example

In this section, the Hepatocellular Carcinoma dataset is modeled using the modified LLR estimators: LLR-ST, LLR-KMW, and LLR-kNNI. Their performances are compared with similar simulation configurations presented in Section 5. The dataset was originally presented by ref. [27] to investigate the gene expression of CXCL17 in hepatocellular carcinoma. Ref. [6] also studied this dataset, comparing parametric and semiparametric models on right-censored data. However, their study focused on a semiparametric model with a univariate nonparametric component using the covariate age. This paper considers a more realistic partially linear additive model (PLAM) that involves two nonparametric covariates.

**Figure 6:**
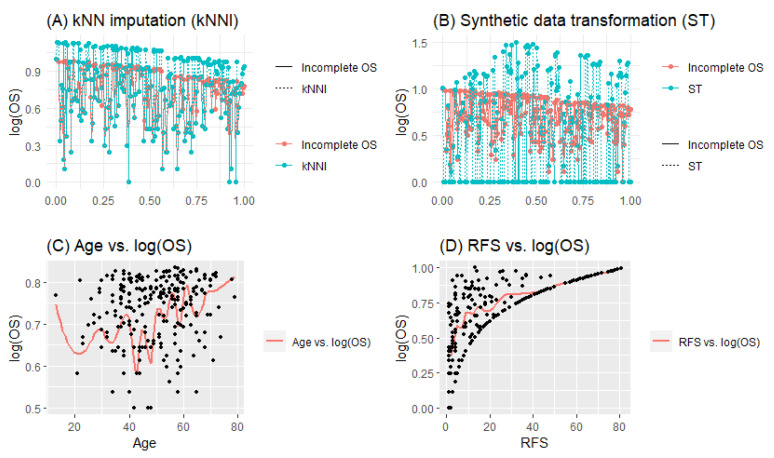
Descriptive plots for the Hepatocellular Carcinoma dataset.

The dataset consists of 227 data points and five explanatory variables: age, recurrence-free survival (RFS), CXCL17T (CXCT), CXCL17P (CXCP), and CXCL17N (CXCN). It should be noted that the logarithm of the response variable, overall survival time (OS), is used in this analysis. The parametric component of the PLAM is determined by the covariates CXCL17T, CXCL17P, and CXCL17N. Additionally, Age and RFS are considered as nonparametric covariates due to their nonlinear structures, as depicted in Figure 6. The figure also illustrates the censored data points versus the transformed data points using the kNNI and ST solutions. Furthermore, panels (C) and (D) display hypothetical curves that represent the data structure and nonlinearity. 

The dataset contains 84 right-censored OS points, indicating a censoring level of CL=37%. This level of censorship can be classified as heavy censoring. Therefore, we expect that the results from the real data analysis may resemble the corresponding simulation configuration of n=200 and CL=35%. Based on the information provided above, the partially linear additive model (PLAM) for the right-censored Hepatocellular Carcinoma dataset can be expressed as follows:

(31) $$\log\left(OS_{i}\right)=\beta_{0}+\beta_{1}\mathrm{CXCL}17T_{i}+\beta_{2}\mathrm{CXCL}17P_{i}+\beta_{3}\mathrm{CXCL}17N_{i}+f_{1}\left(Age_{i}\right)+f_{2}\left(RFS_{i}\right)+\varepsilon_{i\;}$$

where $i=1,\ldots ,227,\;\beta =\left(\beta_{1},\beta_{2},\beta_{3}\right)\;\mathrm{and}\;f=\left(f_{1},f_{2}\right)$. While estimating PLAM in (31), $\log\left(OS\right)$ is replaced by its ST version $\log\left(OS_{\hat{G}}\right)$ and kNNI version $\log\left(OS_{imp}\right)$. Also, KMW is applied. The outcomes of the Hepatocellular Carcinoma dataset with the modified LLR estimators are provided in Table 5.

Table 5 largely confirms the findings of the simulation study and demonstrates the superior performance of the LLR-KMW estimator in the estimation of the parametric component. However, in contrast to the simulation study, the LLR-ST estimator also provides results that are closer to the other two estimators, while the performance of LLR-kNNI is less satisfactory than expected. It should be noted that these conditions may be attributed to the relatively large sample size in terms of censored data. Additionally, regarding the bias of β, as anticipated, both ST and KMW yield lower values compared with kNNI, as they theoretically promise less biased estimates. Overall, the performance evaluation in Table 6 confirms that LLR-KMW exhibits the best results, which are evident from the RE scores.

In both Table 5 and Table 6, the performance of benchmark CPH estimators is also provided and, as expected, it does not show a good performance, especially in the estimation of the nonparametric component. On the other hand, in terms of bias, Table 5 shows that CPH has satisfying bias values but with large variances that cause large SMDE scores. This poor performance is highly related to the lack of the ability of CPH to represent smooth functions. RE scores highly confirm this inference. Summing up the comprehensive assessment presented in Table 6, we encounter an unequivocal affirmation of the preeminent standing of the LLR-KMW estimator. This affirmation is elegantly illuminated by the notable RE scores, reflecting an ensemble of successful estimation endeavors.

**Figure 7:**
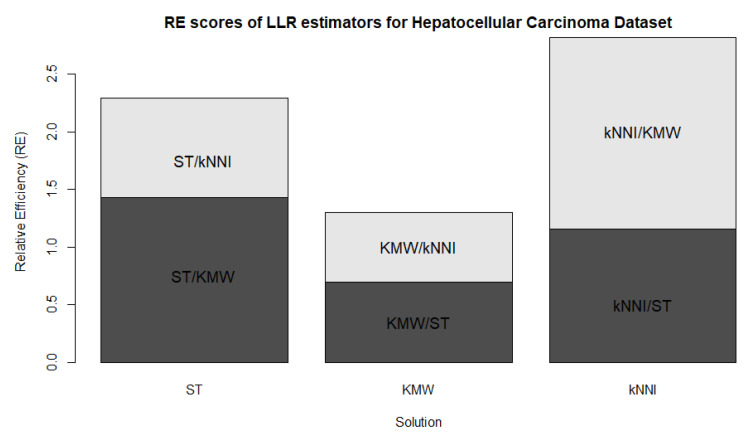
Bar plots of the REs for the modified LLR estimators based on the censorship solutions methods.

In Figure 7, bar plots of the calculated relative efficiencies (RE) are presented. Consistent with the findings in Table 5, LLR-KMW exhibits lower RE scores compared with the other two estimators, which aligns with the results of the simulation study. It is worth noting that while the difference in performance between the estimators may appear significant, numerically they are relatively close to each other, with the RE values scattered around one. 

**Figure 8:**
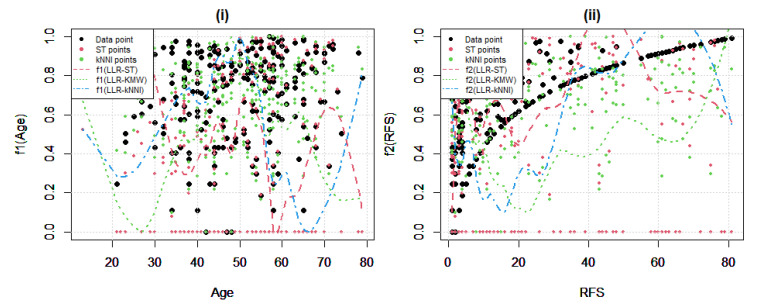
Fitted curves obtained for the Hepatocellular Carcinoma dataset. In panel (**i**) $f\left(Age\right)$ is shown and in panel (**ii**) involves $f\left(RFS\right)$.

After assessing the estimation of the parametric component, Figure 8 presents the results of the estimation of the nonparametric components $f_{1}\;\left(Age\right)$ and $f_{2}\;\left(RFS\right)$. It is noteworthy that in this dataset, the relative failure of LLR-kNNI and the relative success of LLR-ST can be attributed to the structure of the nonparametric components. Both functions $f_{1}$ and $f_{2}$ exhibit favorable structures for the properties of LLR-ST, such as magnifying the magnitudes of uncensored data points and assigning zero to censored ones, as clearly observed in panel (ii) of Figure 8.

To provide a more precise understanding of the solution procedures, the ST points and kNNI points are also included in the plots. These points illustrate why the fitted curves tend to lie below the region where all data points are scattered, especially in panel (ii). This is primarily influenced by the heavy censoring level, CL=37%. Additionally, in panel (i), one can observe the LLR-ST’s fitted curve being pulled down by the zeros. As expected, LLR-KMW follows a balanced approach between the other two estimators, as shown in Table 5, yielding the smallest ARMSE scores in the estimation of the nonparametric component of the PLAM.

## 7. Conclusions

This paper introduces three modified LLR estimators based on different censorship solutions: ST, KMW, and kNNI, to model the right-censored PLAM. For the solution methods that have a theoretical background, such as ST and KMW, the statistical properties and some asymptotic properties of LLR-ST and LLR-KMW are presented. This paper focuses on two main objectives and successfully achieves them. The two purposes of this study are to combine the backfitting LLR estimator with the censorship solutions and to compare them, both theoretically and practically. The performances of the modified LLR estimators are observed through simulation and real data studies. The following conclusions have been drawn from this study:

- In the simulation study, the performance of the estimators is measured individually for both parametric and nonparametric components. Regarding the parametric component estimation, it is observed that LLR-KMW provides the best results, followed by LLR-kNNI. On the other hand, LLR-ST does not yield good results for any simulation configuration, and it is the estimator most affected by the censorship as its performance dramatically changes when the censoring level increases. In this case, LLR-KMW can be considered the most robust estimator, as it reacts to censorship in a more balanced way compared with the other two. In addition, the introduced estimators are also compared with the benchmark estimator for the survival model, CPH. It is observed that the LLR-basis estimators perform better than the CPH, as discussed in Section 6.
- In the estimation of the nonparametric components, the effects of sample size and censoring level are clearly different compared with the parametric component. However, similar to the parametric component, LLR-KMW exhibits dominant performance for both nonparametric functions. It is noteworthy that, as the sample size increases, all three estimators tend to provide closer performances in terms of fitted curves. Furthermore, it should be noted that the performance of the introduced estimators is highly dependent on the structure of the nonparametric component and its compatibility with the chosen censorship solution. Hence, this paper investigates the three different solutions in detail. Ultimately, because the CPH model lacks a smoother structural framework, it falls short when compared with the newly introduced estimators.
- The analysis of the Hepatocellular Carcinoma data serves as a real-world example in this study. This dataset is selected due to its censoring level and sample size, which align closely with one of the simulation configurations (n=200 and CL=35%), enabling a more realistic comparison. The results of the real data modeling demonstrate that the three introduced modified LLR estimators effectively handle the estimation of the right-censored PLAM for both parametric and nonparametric components. They exhibit a good level of agreement with the corresponding simulation configuration, with some minor differences. As expected, LLR-KMW yields the best results. Also, CPH does not show a good performance except in the bias of regression coefficients, as observed in the simulation study. Notably, one important difference between the real data and the simulation study is that LLR-ST exhibits a surprisingly better performance than LLR-kNNI in the estimation of both parametric and nonparametric components. However, this discrepancy can be attributed to the relatively large sample size (n=227), and it does not imply inconsistency with the simulation results. On the contrary, it indicates a close agreement among all performances.

## Figures and Tables

**Table 1:** Calculated SMDE values for all simulation combinations.

n	CL	LLR-ST	LLR-KMW	LLR-kNNI	CPH
50	5%	0.561	0.557	**0.545**	0.991
	20%	0.724	0.681	**0.624**	1.029
	35%	1.084	**0.738**	0.744	1.173
100	5%	0.121	**0.103**	0.104	0.702
	20%	0.140	**0.122**	0.135	0.764
	35%	0.168	**0.142**	0.148	0.834
200	5%	0.027	**0.024**	0.026	0.471
	20%	0.031	0.029	**0.028**	0.480
	35%	0.034	**0.031**	0.033	0.497

Bold color denotes the best performance score.

**Table 2:** Comparative RE scores for the modified LLR estimators.

n	CL	Method	LLR-ST	LLR-KMW	LLR-kNNI	CPH
50	5%	LLR-ST	1.000	**0.992**	**0.970**	1.766
		LLR-KMW	1.007	1.000	**0.977**	1.779
		LLR-kNNI	1.030	1.023	1.000	1.818
		AFT	**0.566**	**0.562**	**0.549**	1.000
	35%	LLR-ST	1.000	**0.686**	**0.680**	1.082
		LLR-KMW	1.456	1.000	**0.991**	1.589
		LLR-kNNI	1.468	1.008	1.000	1.576
		AFT	**0.924**	**0.629**	**0.634**	1.000
200	5%	LLR-ST	1.000	**0.974**	**0.918**	6.333
		LLR-KMW	1.025	1.000	**0.942**	7.125
		LLR-kNNI	1.088	1.060	1.000	6.576
		AFT	**0.158**	**0.140**	**0.152**	1.000
	35%	LLR-ST	1.000	**0.963**	**0.920**	5.794
		LLR-KMW	1.038	1.000	**0.956**	6.354
		LLR-kNNI	1.085	1.045	1.000	5.969
		AFT	**0.173**	**0.157**	**0.167**	1.000

Bold color denotes the best performance score.

**Table 3:** RMSE values of individual nonparametric functions for both functions $f_{1}\left(t_{1}\right)$ and $f_{2}\left(t_{2}\right)$.

	Functions	$f_{1}\left(t_{1}\right)$			$f_{2}\left(t_{2}\right)$		
n	CL	LLR-ST	LLR-KMW	LLR-kNNI	LLR-ST	LLR-KMW	LLR-kNNI
50	5%	0.283	**0.256**	0.260	0.491	**0.473**	0.478
	20%	0.353	**0.241**	0.271	0.535	**0.433**	0.483
	35%	0.447	**0.256**	0.273	0.613	**0.406**	0.479
100	5%	0.383	**0.340**	0.364	0.689	**0.637**	0.668
	20%	0.408	**0.319**	0.366	0.704	**0.581**	0.657
	35%	0.466	**0.323**	0.371	0.754	**0.527**	0.655
200	5%	0.516	**0.483**	0.507	0.936	**0.896**	0.931
	20%	0.537	**0.438**	0.514	0.967	**0.800**	0.927
	35%	0.557	**0.452**	0.517	1.010	**0.727**	0.923

Bold color denotes the best performance score.

**Table 4:** $ARMSE\left({\hat{f}}_{1},{\hat{f}}_{2}\right)$ values for all simulation configurations.

n	CL	LLR-ST	LLR-KMW	LLR-kNNI	CPH
50	5%	0.281	**0.267**	0.271	0.872
	20%	0.319	**0.247**	0.275	0.967
	35%	0.374	**0.233**	0.276	1.008
100	5%	0.393	**0.362**	0.386	0.778
	20%	0.402	**0.334**	0.377	0.814
	35%	0.442	**0.310**	0.381	0.860
200	5%	0.544	0.519	0.539	0.775
	20%	0.565	**0.463**	0.541	0.784
	35%	0.583	**0.438**	0.538	0.841

Bold color denotes the best performance score.

**Table 5:** Performance scores of the introduced three estimators.

	LLR-ST	LLR-KMW	LLR-kNNI	CPH
$Bias\left(\beta_{1};\beta_{2};\beta_{3}\right)$	0.42;0.17;**0.08**	**0.30**;**0.16**;0.17	0.40;0.20;0.21	**0.24**;1.65;0.40
$Var\left(\beta_{1};\beta_{2};\beta_{3}\right)$	0.08;0.26;**0.05**	**0.05**;**0.24**;0.08	0.06;0.26;0.09	0.15;0.68;0.40
SMDE	0.220	**0.154**	0.256	1.341
$RMSE [f_{1}\left(Age\right)]$	**0.440**	0.533	0.491	-
$RMSE [f_{2}\left(RFS\right)]$	0.350	**0.168**	0.208	-
$ARMSE\left(f_{1},f_{2}\right)$	0.395	**0.350**	0.350	1.822

Bold color denotes the best performance score.

**Table 6:** Relative efficiencies; REs.

Estimator	LLR-ST	LLR-KMW	LLR-kNNI	CPH
LLR-ST	1.000	**0.699**	1.160	6.095
LLR-KMW	1.429	1.000	1.659	8.707
LLR-kNNI	**0.861**	**0.602**	1.000	5.238
CPH	**0.164**	**0.114**	**0.190**	1.000

Bold color denotes the best performance score.

## Data Availability

The Hepatocellular Carcinoma dataset is publicly available in R-package named “asaur”.
